# Hospital and Institutional News

**Published:** 1914-06-27

**Authors:** 


### HOSPITAL AND INSTITUTIONAL NEWS.

### THE BIRTHDAY HONOURS.

Institutional workers will learn with interest that among the knights included in the Birthday Honours is Dr. John James Burnet, LL.D., F.R.S., who, in conjunction with Dr. Mackintosh, M.V.O., also of Glasgow, is the architect of the Glasgow Royal Hospital for Sick Children, which is to be opened almost at once, and which is stated to begin a new era in the design of children's hospitals. Sir James Burnet is also architect of the new King Edward Galleries at the British Museum. A knighthood is also given to Mr. James Bradford, who has built and endowed a hospital and almshouses at Hayward's Heath. Another institutional enthusiast who also receives a knighthood is Mr. James E. Jones, the chairman of the Manchester Royal Deaf and Dumb Schools, who has much interested himself in the education of these unfortunates. Passing now to the medical profession, knighthoods have been conferred upon Mr. Wilmot Parker Herringham, M.D., F.R.C.P.; Mr. William Milligan, M.D.; and Mr. Seymour John Sharkey, M.D., F.R.C.P. Sir Wilmot Herringham is physician to St. Bartholomew's Hospital and Vice-Chancellor of London University. Sir William Milligan, who is aurist and laryngologist at the Royal Infirmary, Manchester, and surgeon to the Manchester Ear Hospital, was educated at Aberdeen, Gottingen, and Vienna, and is a well-known publicist on his speciality. Sir Seymour Sharkey, medical referee to the Treasury and consulting physician to St. Thomas's Hospital, himself the son of a medical man, was a classical scholar at Oxford before adopting the profession of medicine, and there he has acted as examiner in pathology and medicine. He has also been censor to the Royal College of Physicians. The K.C.B. is also conferred upon Surgeon-General Arthur William May, C.B., K.H.P., Director-General of the Medical Department of the Royal Navy.

### MEDICAL MEN AND THE COLONIAL LIST.

The honour of Knight Bachelor has been conferred upon Mr. Thomas George Roddick, M.D., a medical practitioner of Montreal, and on Mr. Thomas Peter Anderson Stuart, M.D., who has been Dean of the Faculty of Medicine at Sydney University for thirty-one years. He is regarded in Australia as the best-known university professor in the Commonwealth. A similar honour is also bestowed upon Mr. Alfred Edward Thomson, of Cape Town. The distinction of the C.M.G. falls to Mr. Frank Gerard Clemow, M.D., the British Delegate to the Ottoman Board of Health. Lieutenant-Colonel William Rice Edwards, C.M.G., M.D., of the Indian Medical Service, and Colonel Courtenay Clarke Manifold, M.B., of the Indian Medical Service, receive the C.B., while the C.I.E. is bestowed on Lieutenant-Colonel William Molesworth, M.B., B.S., M.R.C.S., L.R.C.P., of the Indian Medical Service, Surgeon, 1st District, and Superintendent, Medical School, Rayapuram, Madras; on Lieutenant-Colonel George James Hamilton Bell, M.B., the Inspector-General of Prisons, Burma; and Major Edward David Wilson Greig, M.B., B.Sc., Assistant Director, Central Research Institute, Kasauli. Lieutenant-Colonel Leonard Rogers, C.I.E., M.D., F.R.C.P., F.R.C.S., Professor of Pathology, Medical College, and Bacteriologist to Government, Calcutta, receives a knighthood; and the Kaisar-I-Hind Gold Medal is awarded to Major Charles Edward Southon, M.B., Indian Medical Service, Chief Plague Medical Officer, Punjab; and to Honorary Captain William John Alexander Hogan, Indian Subordinate Medical Department, Civil Surgeon and Superintendent of the Jail, Muzaffarnagar, United Provinces. Finally, Mr. Harold Robert Dacre Spitta, M.D., Bacteriologist to his Majesty's Household, is appointed M.V.O., fourth class. It will be seen at once that the above list offers nothing of unique interest to the institutional and medical world; merit has been rewarded, but some of those heroic workers, whose claims we have more than once alluded to, are still passed over, and, it may be noted in conclusion, the House of Lords remains without a medical peer.

### ROYAL INFIRMARY, LIVERPOOL.

We had an opportunity of seeing the new tropical diseases ward and laboratory at the Royal Infirmary, Liverpool, on the 22nd instant. These additions will strengthen the educative and scientific value of the work done at this hospital, and their plan of construction promises well. Neither is at present finished, and a full notice must wait their completion and opening for work. We availed ourselves of the opportunity offered to visit the new out-patient department during working hours (see The Hospital, September 10, 1910, pages 718, 719). The inspection gave us infinite pleasure, for this unit, which is the latest addition to the Liverpool Royal Infirmary, is undoubtedly the perfected unit for an out-patient department so far as modern hospital construction is concerned. It is an admirable unit, well planned and artistically carried out. Its only drawback yet manifested is some defective acoustic properties of the central hall, which would have been prevented had due weight been given by the architect to the warnings he received from a member of the staff who is a technical hospital expert.

### RENEWED STAFF TROUBLE AT CAMBERWELL.

The Camberwell Board of Guardians are again faced with serious trouble in connection with their medical staff. Not many months ago they had to place certain assistants on probation for three months for alleged insubordination, the doctors alleging they were subjected to treatment which no child would submit to. They have left the service of the Board since that date, many of them being granted testimonials of the highest character, and one allowed extra leave, ere they left. Now, in their place in the last six months has been appointed an entirely fresh staff, who, in turn, have gone. The situation was described by Dr. Keats, the medical superintendent, in a report presented to a special meeting of the Guardians. In this Dr. Keats states that at 3.35 on the morning of the 12th inst., in consequence of the disturbance to the institution, owing to the noisy talk and singing from the assistant medical officer's sitting-room, he visited the room, and was " disgusted at the sight " presented to him. " Drs. Hill, Warburton, and Pearson were sitting there at a table with a male friend, and on the table were several empty bottles of beer. They were told by me that such a sight, such noise, as they had been making was a disgrace to a public institution, and that although I had seen rowdyism I had never known anything quite like that before. Dr. Warburton said ' It is all right, I am giving in my notice in the morning.' Drs. Hill and Pearson each said ' It is all right, sir, there is nobody making any noise here.' "

### THE MEDICAL OFFICERS AND THE COMMITTEE.

Dr. Keats said he requested the visitor to leave, and advised the others to go to their bedrooms quietly and without delay, but instead at four o'clock with their friend the three doctors left the infirmary. They returned the next morning, and did part of their duty, eventually leaving a note for him stating they were resigning. Interviewed by the visiting committee, Dr. Hill said they were " told to go to bed like children, and that he and the two other assistants left the infirmary as a protest against the petty rules of the institution." Dr. Warburton told the committee that he " claimed the right to make a row in the sitting-room, that the rules and regulations of the institution were ridiculous, and that he refused to obey some of them." The committee decided to suspend the doctors, and reported the whole matter to the board, who were called upon to decide what action should be taken. The doctors were invited to attend, but wrote declining to do so, because they had handed in their resignations, and therefore no useful purpose would be served by attending the board meeting. They added that if the board wanted any information they should invite the Local Government Board to hold a public inquiry into the administration of the infirmary.

### THE GUARDIANS' DECISION.

The board discussed the matter for half an hour in private, when the Chairman proposed that the board dismiss the three medical officers forthwith, and pay them up to that date, and that the decision, together with all the facts that had been placed before them, be forwarded to the Local Government Board, the doctors to leave the institution before twelve o'clock the next day. It was also decided to add the words " for gross insubordination " after the word " forthwith." This was agreed to by all the Guardians present, with the exception of the Countess de Lormet, who did not vote. From the frequent disturbances that have occurred at the infirmary, it is evident that even if the Local Government Board does not intervene, as they may not, seeing that the doctors have left, the Guardians are in duty bound to see where the trouble arises in the interests of the patients, of the board, and of the ratepayers, who will be called upon to pay the extra salaries for temporary aid. In other institutions there may be slight trivial troubles from time to time, which are quickly surmounted, but they never cover the extended period as in the case at Camberwell. Hence the greater reason for an exhaustive inquiry into the causes of the trouble.

### THE DEVELOPMENT OF THE WARD FUND.

In most institutions there are one or more ward funds for various purposes. " A suggestion, which might be of interest and service to institutional workers," writes a correspondent, " is that of a ' plant fund,' which is known to be in existence in at least one provincial institution. The patients are asked to contribute a penny or two each, and the money thus collected is used for the purchase of suitable pot plants or cut flowers for the wards. Though it must be obvious that at certain seasons of the year hospitals are well-nigh overdone with flowers, yet since there are other times when floral decorations are at a premium the above suggestion might be of real utility." The difficulty presented by this proposal is that hospital managers, now that patients' contributions are increasingly common, will be unwilling to divert this source of income to any minor ends, however desirable, for fear the feeling should grow that " everything has to be paid for: even the flowers they give us in the wards are charged for," and so on. But there is no reason why the common practice of patients giving donations for the special benefit of the ward they have been in should not be used not only for crockery and ward articles, but also expended on flowers. The whole question of ward funds and crockery was discussed in The Hospital of November 22, 1913, p. 200; but as regards flowers, it is probably better to encourage the gift of them from hospital visitors.

### VOLUNTARY BOARDERS AT REGISTERED HOSPITALS.

The difference between insanity and mental disease has always appeared to be that the former was a legal matter and the latter a medical one. A medical man could diagnose some form of mental disease and treat the patient for it at home, but if any institutional treatment had to be resorted to the patient had to be legally declared to be a " lunatic." This has always been the point where great care has to be exercised and where the objections of the patient's friends arise. True, private patients suffering from mild and early symptoms can voluntarily place themselves under care without a magistrate's order, if they can find a place within their means to take them in, but such places are few. The pauper patient has never had such facilities, however, and the sceptics doubt if they would take advantage of them even if they existed. Anyone who has worked amongst the pauper insane will realise that there are very many of them who not only realise their own disabilities, but are anxious to place themselves under care when they feel they are in danger of a breakdown. It is these cases, too, that are most amenable to treatment and who best repay any effort made on their behalf. At one of the registered hospitals with which we are acquainted voluntary boarders began to be admitted a few years ago, and the result was certainly surprising. The average admission rate of certified patients was between eighty and ninety a year, and this average has been maintained; but as soon as the system of admitting voluntary boarders was fully introduced the average number so admitted became nearly forty a year.

### THE ECONOMIC FACTOR.

These consisted of former patients who came back for treatment at the earliest signs of any symptoms, and new patients who had never been certified. The great majority recover, some go out relieved, and a few develop such symptoms as to render certification necessary. The most striking point has been the economic one. Those who go out recovered do so in half the time that the certified patients do, thus saving half the cost of maintenance in the institution, and getting back to work as useful members of the community in half the time. Judging by this experience, it would seem probable that the sceptic is a false prophet, and that the pauper patient who is best worth helping would take advantage of the chance offered him.

### HOSPITAL BEDS FOR THE LONDON POLICE.

Dr. Maurice Cassidy, one of the assistant physicians at St. Thomas's Hospital, it is announced in the *Lancet*, has been appointed physician to the Metropolitan Police Force. This is a new departure, on which the governing powers of the Force are greatly to be congratulated. Hitherto the medical care of the London constables has been confided to the chief surgeon (Mr. Ballance) and his staff of district surgeons, who are, of course, general practitioners. There has been no official cognisance of the need for a physician, though in practice the interests of the Force have not been allowed to suffer thereby. The district surgeons are just as well qualified to treat medical cases as surgical ones, and the chief surgeon has usually been some broad-minded senior consultant who could be trusted to appreciate the medical problems presented to him, and to obtain the services of expert hospital physicians when necessary. This was particularly true of the late chief surgeon, Mr. Clinton Dent; and this new appointment shows that Mr. Ballance also is impressed with a sense of the necessity. In expressing approval of Dr. Cassidy's appointment, our contemporary suggests that special beds should be set apart for the Police Force in the great Metropolitan hospitals. Fully admitting that our constables deserve the very best that the institutional world can give them, we yet consider that such an arrangement of reserved beds would be a mistake, because in the nature of things it would surely happen that the demand for the beds would vary from time to time, thus involving a waste of the resources of the institutions concerned.

### NEW PATHOLOGIST AT THE CHEST HOSPITAL.

Dr. W. E. Carnegie Dickson has just been appointed to the post of Director of the Pathological Department and Research Laboratory of the Royal Hospital for Diseases of the Chest, City Road. Dr. Carnegie Dickson is at present pathologist and bacteriologist to the Royal Hospital for Sick Children, Edinburgh, and Lecturer in the University of Edinburgh. He has held several research scholarships in Edinburgh, where he graduated in 1901. He has been a Fellow of the Royal College of Physicians, Edinburgh, since 1908. Dr. Dickson has done a great deal of pathological work, but is particularly known for his thesis on the bone marrow and its cytology, for which he was awarded a gold medal in 1905, and which he presented for his M.D. The text-books on pathology for students, which Dr. Dickson wrote in collaboration with Professor J. M. Beattie, have become standard works, and are now in their second editions. By this appointment the management of the Chest Hospital have shown their anxiety to make full use of the clinical material at the Royal Chest Hospital, and it may be hoped that the new pathologist will be able to elucidate some of the problems as yet unsolved in connection with the study of tuberculosis of the lungs.

### TUBERCULOSIS OFFICERS AND COMBINATION.

In a previous issue we had noted an evident desire for some sort of professional union on the part of tuberculosis medical officers, and had intimated—a fact which did not seem to be as widely known as might be expected—that a body known as the Tuberculosis Society already existed to fulfil the desired object, and had a good membership. Last week we printed a letter from a tuberculosis officer to a large area in Yorkshire, mentioning that meetings in London were inconvenient to workers in the North, criticising one or two minor details of the constitution of the society, and reiterating a suggestion that the July annual conference of the National Association for Prevention of Consumption, to be held in Leeds, would be a good opportunity, could members of the existing executive committee of the Tuberculosis Society attend, to discuss these points. If this suggestion does not commend itself to the executive it will be unfortunate, because, of course, from the existing distribution of population in England, the area from Sheffield to the Tyne must possess a great claim to consideration. Indeed, two county administrative areas alone—namely, Lancashire and the West Riding of Yorkshire—have more than twenty tuberculosis officers, while the county boroughs contained here, with those further north, bring up the total to a number much larger than in the London and home counties district—larger, indeed, than any area of equal size in the United Kingdom. A young society, then, which should surely encourage that natural faculty of youth—namely, growth—might well welcome an occasion to strengthen itself. Already it has enrolled amongst its members a good proportion of the present maximum obtainable, but in spite of this there is no doubt at all as to what would constitute good, and what bad, tactics in the present connection. But perhaps, if our correspondent be successful in convening the informal meeting that he proposes, then a well-signed memorial embodying some of the points mentioned above would receive the attention which such an important expression of sectional opinion must merit. There appears to be no positive obstacle to provincial meetings inherent in the society's constitution. Rule 12 merely says that the place and date of the meetings shall be decided by the committee; and recently, we believe, one was fixed to be held at Oxford.

### SOLIDARITY IN TUBERCULOSIS WORK.

In the interests of the new public medical service, of such rapid recent growth, it would be a good thing could other outlying areas make a similar move towards promoting a lasting solidarity. As regards the form now under notice—namely, anti-tuberculosis work—Dr. Paterson's well-known organising powers would soon find the methods best suited in the case of Wales, while a prominent tuberculosis officer in the West of England, who seems, from a letter to a contemporary, to be interesting himself in "the more efficient and unified organisation of central and local administrative departments and executive services of public health," might find in the present connection a useful subject for his attention. Of the highly important case of Scotland we say nothing, because the Tuberculosis Society possesses the advantage of Sir R. W. Philip's advice as Honorary President. Ireland, more inaccessible geographically, has already a society of its own. Moreover, those who have read reports of the yearly assemblies of the kindred association in Germany, the association of *Tuberkulose-Aerzte*, will have recognised and found in them most useful first-hand information. If the spirit and level of attainment apparent between the green-paper covers of these *Berichte* were striven after, the Tuberculosis Society would effect much good to all directly or indirectly concerned.

### SUDDEN DEATH OF A HOSPITAL SURGEON.

The Bristol Eye Hospital will regret the loss by death of one of its honorary consulting surgeons in the person of Dr. Alexander Ogilvy, which occurred quite suddenly while he was preparing to see some of his out-patients. Dr. Ogilvy, who was an M.D. and F.R.C.S. (Ireland), was held in the highest esteem by his colleagues at the Eye Hospital, as well as at the Royal Infirmary, where he was also a member of the staff. His loss to institutional life in Bristol will be a real one.

### MENTAL DEFICIENCY WORK IN LONDON.

The Mental Hospitals and Mental Deficiency Committee have informed the London County Council that it is necessary that a woman inspector should be added permanently to the staff of the Public Health Department to assist in that part of the work under the Mental Deficiency Act which is to be carried out under the supervision of the medical officer of health. The person appointed should have the qualifications of a health visitor, with nursing experience in a mental hospital or an institution for mental cases. Applications for the position are to be invited by public advertisement, the salary attached to the office being £150 a year.

### THE CREMATION SOCIETY.

It is always interesting to record the progress which cremation makes in this country, for if slow it is steady, and therefore indicative of a positive growth in popularity. Towards consolidating this support the existence of the Cremation Society certainly provides a useful means, for by membership is conferred the right to guarantee cremation to those in favour of this mode of disposing of the dead, so far as it can be guaranteed under proper conditions and regulations. It is fortunately beginning to be recognised on a wider scale that the problem is not a sentimental one—in fact the pressure on our existing burial grounds is now so great, and the difficulty of extending them sufficiently to meet the demand is now becoming so general, that the practical social advantages offered by cremation are more and more impressing themselves, slowly but insistently, upon the public mind. Hitherto, or till lately, the advocates of cremation have really relied upon the advertisement given to their cause by the preference of well-known personalities to drive home its advantages to the outside public. Sir Henry Thompson will always be remembered in this way, and we note that the society still finds it advisable to print "a list of persons of note" who have been cremated at Woking and elsewhere. To the student of human nature alone this list provides interesting reading: a collection of names which, for the most part, would seem to have had no possible common bond is here shown as those united in preference for cremation over earth burial. We may note, by the way, in passing, that though this list is described as abridged, it does not contain the name of a man whose genius is probably becoming more widely recognised than that of any recent English scientist—namely, the late Samuel Butler, who was cremated at Woking about twelve years ago. If it is worth the Society's while to print a list of people of note who have been cremated, it is surely worth its while to include that of Samuel Butler, and the omission is extraordinary in an otherwise interesting and well-thought-out report.

### DEATH OF A VETERAN HOSPITAL CHAPLAIN.

We regret to have to record the death of the Rev. Henry James Fase, M.A., which has occurred after a short illness. Mr. Fase had been for thirty-two years chaplain to the Westminster Hospital, and it was only a matter of a few months ago that we recorded in these columns his resignation of the post and his retirement from active work at the hospital he had served so long.

### THE SURPLUS PANEL FUND AND THE LONDON INSURANCE COMMITTEE.

The question of the distribution of the surplus panel fund—amounting to about £90,000—was again discussed by the London Insurance Committee at its meeting on June 25, when a further report on the matter from the Medical Benefit Sub-Committee came up for consideration. It will be remembered that at last month's meeting of the Committee the recommendation of the Medical Benefit Sub-Committee that the money should be distributed according to Article 40 of the Medical Benefit Regulations was referred back, and now, after reconsidering the matter, the Sub-Committee reports that it is unable to recommend a definite course of action, but is of opinion that the Committee should decide either (1) to authorise payment to each practitioner on the panel during the year ended January 11, 1914, of an amount bearing the same proportion to the sum credited to him as the amount on the Panel Fund for such year . . . bears to the amounts credited to all the practitioners; or (2) leave the distribution of the surplus fund with the Insurance Commissioners. The Medical Benefit Sub-Committee reports that eight resolutions have been received from various bodies protesting against the distribution of the surplus fund amongst the practitioners on the panel, and also reports the receipt of a letter from the Commissioners, in which they adhere to the opinion that the Medical Benefit Regulations preclude the exercise of any discretion on the part of an Insurance Committee to vary the method of distribution.

### GLASGOW CHAIRMAN'S FINE RECORD.

Some two months ago Mr. James D. Hedderwick, who is resigning the chairmanship of the Glasgow Royal Infirmary after ten years' tenure of it and twenty-two years on the Board, was presented with his portrait by past and present members of the Board in recognition of his long and distinguished services to the infirmary. During the ten years of his chairmanship the great task of reconstruction has been faced and successfully carried through; and it is due in large measure (says the *Glasgow Medical Journal*) to the ability, energy, tact, and unremitting industry of Mr. Hedderwick that the labours of the Board of Management have been brought to so admirable a conclusion. Furthermore, it is a great pleasure to add that the University has decided to express its indebtedness to Mr. Hedderwick in its own separate way—namely, by granting him the honorary degree of LL.D. His name is known as one of the ablest hospital chairmen of the day, and these recognitions of his work are no more than his deserts. Mr. Hedderwick is also an ex-President of the Glasgow Chamber of Commerce.

### PHARMACISTS AND THE BRITISH PHARMACOPŒIA.

The new edition of the British Pharmacopœia, of which a proof was placed on the table at the recent meeting of the General Medical Council, is the last edition in the compilation of which the Pharmaceutical Society will co-operate on the present terms. The President of the Pharmaceutical Society has, in fact, publicly intimated that the Pharmaceutical Council has resolved that after the publication of the forthcoming edition "it will no longer be prepared to provide material and the assistance of its staff for any future editions without material alteration of the existing conditions." Since the passing of the Medical Act of 1858 the General Medical Council has been solely responsible for the production and revision of the Pharmacopœia, and although the assistance of pharmacists has always been available, the Pharmaceutical Society has never had a recognised status in connection with the production of the official work. In the preparation of the forthcoming edition the Committee of Reference in Pharmacy has, in fact, done most valuable work, and it is difficult to imagine what a Pharmacopœia would be like if it were produced without the assistance of pharmacists. The General Medical Council, however, holds the view that under the Medical Act they are compelled to relegate the production of the Pharmacopœia to a committee composed of medical men only, and if this view is correct it is obvious that before a subsequent edition can be produced some amendment of the statutory conditions will have to be made if the services of pharmacists are to be retained.

### THE ORIGINATOR OF HOSPITAL SUNDAY.

The Lord Mayor of Newcastle, speaking at the British Hospitals Association Conference on June 18, is reported to have declared that the late Alderman Richard Henry Holmes, of Newcastle, was the originator of the Hospital Sunday Fund forty-five years ago—that is, in 1869. It may be well, therefore, in support of historical accuracy, to point out that Hospital Sunday was founded in Birmingham by the late Canon Miller and Mr. T. B. Wright in 1859, or ten years before the movement is stated to have first commenced in Newcastle by the late Alderman Holmes. Those of our readers who have a file of The Hospital, or who preserve as a reference library all the volumes of "Burdett's Hospitals and Charities," will find a detailed history of the Hospital Sunday movement in vol. x. for 1899, vol. xxiv. for 1913, and vol. xxv. for 1914, chapter iv.

### THE ADMISSION OF PATIENTS.

Voluntary hospitals have grown so long accustomed to heavy waiting lists that it is only of late that the urgent necessity of dealing with the problem on an adequate scale is becoming practically recognised. No doubt the Insurance Act, which has increased the number of in-patients, partly because there is a temptation to panel practitioners to send every case they can for hospital treatment, has done much in this way to bring the question forward, but also, as the approved societies have begun to find, it is not only malingering, but the more adequate tabulation of actual disease, which accounts for the drain on their funds, and therefore for the number of patients. It is a symptom of the times, then, that Mr. Sherman

Bigg should suggest in the *Lancet* that "the rules for the admission of patients to hospitals need revision" in order to prevent the refusal of suitable cases. There is no doubt something in this view, but the provision of further accommodation is the root of the problem, and it is one which inevitably came up for incidental discussion at the Conference of the British Hospitals Association, which we report elsewhere. We believe its solution will be found not by the, to some, inviting theory of municipalisation, but rather on co-operative lines; since one factor at least in our present difficulties in this respect is that in various public institutions all the available beds are not made use of, or, again, expensive hospitals are built only to be used for chronic cases. Facts like these point to a stock-taking of our existing resources rather than to any far-reaching and extremely costly scheme on purely municipal lines. Co-operation in London has achieved much for sanatorium patients; why can it not be extended to the country and other than tuberculous cases as a whole?

### IMPORTANT EXTENSION OF LONDON MENTAL HOSPITAL WORK.

An important project for increasing the accommodation available for mental patients is announced by the London Mental Hospitals and Mental Deficiency Committee. They report that an offer has been received from the owner of Portnalls, a property adjoining the Cane Hill Mental Hospital Estate, to sell a portion of the property of not less than 72¼ acres of land with a house at a maximum price of £15,000, with a reduction at the rate of £150 an acre if on survey the land is found to be of less extent. It is pointed out that Cane Hill Mental Hospital has a very small estate, the area of which is only 155 acres, and this, though probably sufficient for the requirements of the 1,200 patients originally accommodated, is not adequate for the 2,200 patients housed there now, there being more than fourteen patients to each acre of land. In the case of a new mental hospital the instruction of the Board of Control as to the sites is that "the quantity of land to be acquired . . . should allow the proportion of not less than one acre of land to every ten patients which the mental hospital will ultimately contain." If the Portnalls property is added to the estate the result will be a compact area, rectangular in shape and completely isolated by public roads from other property. The additional land, which is practically all pasture and arable, will be of great service, as the present acreage available for agriculture is extremely limited, and there are only eight acres of grazing land for sixty cows. If the property is not purchased there is a probability of its development for residential premises, and in that event a substantial barrier, which has not been needed hitherto, would be required to isolate the mental hospital area.

### THE PORTNALLS ESTATE.

The house, which is offered with the land, could be adapted immediately, at a cost of about £2,300, to provide accommodation for some fifty patients—an important consideration at the present moment when it is necessary to send London patients to other counties. If in future the provision of additional accommodation in this locality should be found necessary, the Portnalls Estate would furnish a possible site for additional villas to be administered as part of Cane Hill Mental Hospital, or even, severed from the present Cane Hill Estate, a site for a separate institution for some 700 patients. The County Council are being urged to buy the estate, and a provisional contract for sale has been entered into with the owner. The proposal to acquire the property has the support of the Board of Control, who are prepared to recommend the Home Secretary to approve its purchase.

### THIS WEEK'S DRUG MARKET.

The improvement in the tone of the market noticed last week has not been fully maintained, and, with the exception of a brisk demand for such articles as tartaric acid and citric acid, which are usually in strong request at this season, business has been rather quiet. Tartaric acid tends upwards in price, and there are no indications of the probability of any decline in the value of citric acid, which continues exceedingly scarce. Cod-liver oil is in very small demand, and the price is barely maintained; any substantial alteration in value, either upwards or downwards, is hardly expected, and as far as can be foreseen the price seems likely to remain round about the present quotations, at any rate for a little time. The scarcity of nux vomica has become more pronounced, and, consequently, a further advance in the price of strychnine is probable. There has been no further change in the price of camphor. The price of opium has an upward tendency, and the same may be written of morphine. As yet there is no change in quotations for quinine. Essence of lemon is again rather higher in price, and it is difficult to foresee the future of this article. The position of cocaine is unchanged.

### "THE HAMPSHIRE TUBERCULOSIS OFFICERSHIP."

Our attention has been called to the following paragraph, which formed a portion of the leading article on page 282 of The Hospital of June 13, 1914: "That tuberculosis work does nowadays require special experience and training is recognised in these application forms, which require of candidates particulars of such. It was also recognised a year or two ago by their present medical officer of health, who, as was then brought to our notice, circularised sanatorium superintendents with a request for information about the use of tuberculin." We have received evidence, which we accept as authentic, that Dr. Lyster, the present medical officer of health to the Hampshire County Council, "has not sought information from sanatorium superintendents *re* tuberculin," and that the statement is consequently "without any foundation whatever." We accept this disclaimer and express our regret that the article should by inadvertence have contained the statement in question, which we unreservedly withdraw.

